# Application of Machine Learning in Air Hockey Interactive Control System

**DOI:** 10.3390/s20247233

**Published:** 2020-12-17

**Authors:** Ching-Lung Chang, Shuo-Tsung Chen, Chuan-Yu Chang, You-Chen Jhou

**Affiliations:** 1 Department of Computer Science and Information Engineering, National Yunlin University of Science and Technology, Yunlin 64002, Taiwan; chang@yuntech.edu.tw (C.-L.C.); chuanyu@yuntech.edu.tw (C.-Y.C.); shough33@gmail.com (Y.-C.J.); 2 Bachelor Program in Interdisciplinary Studies, College of Future, National Yunlin University of Science and Technology, Yunlin 64002, Taiwan; 3 Artificial Intelligence Recognition Industry Service Research Center (AIR-IS Research Center), National Yunlin University of Science and Technology, Yunlin 64002, Taiwan

**Keywords:** AI, machine learning, convolutional neural network, YOLO, linear guideway, stepper motor, air hockey game

## Abstract

In recent years, chip design technology and AI (artificial intelligence) have made significant progress. This forces all of fields to investigate how to increase the competitiveness of products with machine learning technology. In this work, we mainly use deep learning coupled with motor control to realize the real-time interactive system of air hockey, and to verify the feasibility of machine learning in the real-time interactive system. In particular, we use the convolutional neural network YOLO (“you only look once”) to capture the hockey current position. At the same time, the law of reflection and neural networking are applied to predict the end position of the puck Based on the predicted location, the system will control the stepping motor to move the linear slide to realize the real-time interactive air hockey system. Finally, we discuss and verify the accuracy of the prediction of the puck end position and improve the system response time to meet the system requirements.

## 1. Introduction

Advances in integrated circuit (IC) design technology have made significant contributions in both memory space and processing power. This has boosted the feasibility of artificial intelligence technology applications [1]. How to combine machine learning with machine control is an important research issue.

Related works usually got the position of the puck by traditional image recognition. The mutual conversion of the color space is used to eliminate the influence of the brightness on the color characteristics of the puck [2]. The prediction first assumes that the puck moves in a straight line, and a linear formula is used as the prediction method after the last impact on the edge of the table or after the puck enters the specified area [3]. It was also proposed to use a neural network for the end point. Position prediction [4] uses the angle, speed, and position of the puck through the attack line as input data to train a neural network model that can predict the position of the puck through the finish line. Compared with directly extracting the puck position for trajectory prediction, some have proposed adding a high-speed vision system to capture the player’s attacking postures and movements to make the basis for the robot’s return decision [5], or to analyze the eye movements of human players to generate a return strategy using the human eye to determine the lack of time [6], and some people make a control mechanism based on reinforcement learning to reduce the rate of missing points of this system [7].

Due to the development of convolutional neural networks, many people began to apply neural networks to image recognition [8,9,10,11,12,13,14,15,16,17,18,19,20,21]. The currently developed target detection algorithms are divided into two types. The first type is to divide the detection problem into two stages. The first is to generate regional proposals and classify these regions, for example: region convolution neural network (R-CNN), fast R-CNN, faster R-CNN [22,23,24]; this type of detection model has a low error rate, but it takes a long time to execute. The other type is to directly predict the probability and position of the target type, such as “you only look once (YOLO)” and “single shot multibox detector (SSD)”; this type of model has a faster detection speed. YOLO is an algorithm that uses target detection as a regression problem. It gets features by a convolutional neural network, and gets bounding boxes and confidence score by a fully connected layer, split image into S × S grid, and predicts bounding boxes in every grid. Each bounding box contains position, size, and confidence score. After modification and optimization, it removes the fully connected layer [25] and uses multiple independent regression classifications to solve the multilabel target classification, also improving the detection of small objects by multiscale output dimensions. The prediction of the bounding box also refers to anchor boxes in faster R-CNN, these anchor boxes are decided by K-means [26]. There is a lightweight network model, compared with the original model; although some precision is sacrificed, the speed is much improved.

In this work, we mainly utilize deep learning combined with motor control to realize the real-time interactive system of air hockey, and to verify the feasibility of machine learning in the real-time interactive system. The air hockey game is a competitive entertainment game that combines intelligence and reaction ability. It consists of a table with a goal on each side. The surface of the table has evenly distributed air holes, and the fan under the table produces air flow from these holes. They allow the air hockey puck to slide on an almost frictionless table. The puck is a plastic disc. Player uses the plastic handle to hit the puck into the opponent’s goal and not allow the opponent to score as much as possible. The trajectory of the puck will be in a linear motion, but in reality, it will not be such an ideal situation. The friction of the tabletop and the levelness of the table will affect the trajectory and the final position of the puck. Thus, a camera is set up on the air hockey table, the real-time image is captured and connected to a personal computer (PC) via a USB interface, and the position of the hockey puck in the image is identified using the YOLOv3 convolutional neural network. At the same time, it uses the law of reflection and neural network to predict the possible final point of the hockey. The feedback controls the stepper motor to move the linear guideway to the possible final point of the hockey.

The rest of this paper is organized as follows. Section 2 reviews some preliminaries. Section 3 introduces the proposed system architecture. Section 4 presents experimental environment and results. Finally, Section 5 provides a conclusion.

## 2. Preliminaries

**Figure 1:**
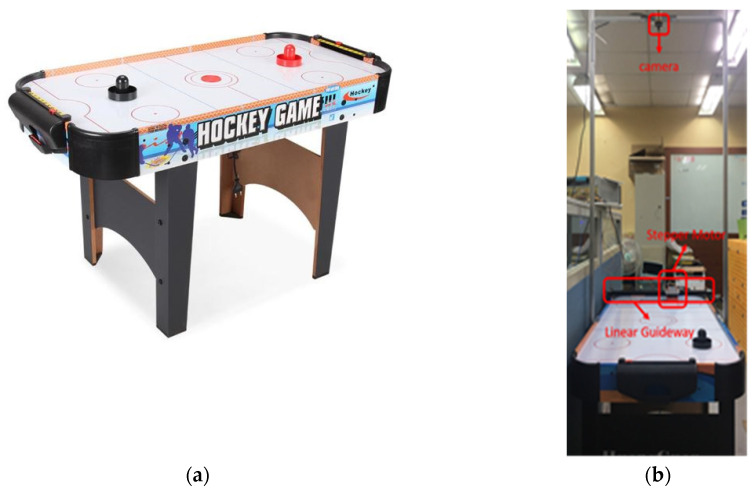
(**a**) Full picture of an air hockey table. (**b**) The air hockey table is set up with a stepper motor, a linear guideway, and a camera above.

Air hockey is a competitive entertainment game that combines intelligence and reaction ability. As shown in Figure 1a, the game environment consists of a table with a goal on each side of the table. The surface of the table has evenly distributed air holes. The fan under the table will generate airflow from the air holes, allowing the plastic disc, hockey puck, to be almost sliding on a frictionless tabletop. The player uses the plastic handle to try to penetrate the opponent’s goal and block the opponent’s attack as much as possible.

The linear slide rail in air hockey is a rolling guide, using steel balls or rollers as the transmission interface between the slider and the slide rail to carry out rolling motion without limit of stroke, so that the load platform can follow the slide rail at high speed, high-precision linear motion. Due to the low frictional resistance, the drive horsepower is low, and the requirements for miniaturization and high-speed machinery can be realized at the same time.

The stepping motor in air hockey is a control element that converts electrical pulse signals into angular displacement or linear displacement. When there is no overload, the speed and stop position of the motor depends only on the frequency and number of pulses of the pulse signal. The influence of load changes, and the stepper motor has only periodic errors without cumulative errors. The stepping motor has a stator and rotor that are like gear-like protrusions and fit each other, and the current flowing to the stator coil is switched to gradually rotate at a certain angle. The stepping motor has a great self-retaining force when it generates a magnetic field after being energized, so it can maintain the stopped position even without using a mechanical brake. Although the stepping motor is small in size, it can achieve high torque output at low speed, so it has good performance in acceleration and frequent movement.

By setting up a camera on the air hockey table (shown in Figure 1b), the real-time image of the hockey position can be captured and transmitted to a personal computer (PC). In order to block the hockey smoothly, the defender must move to the corresponding position before the hockey goal is scored. Therefore, it is necessary to obtain the position of the hockey and predict the hockey end position in advance, and finally move the defender according to the predicted result.

## 3. Proposed System Architecture

This section presents the proposed system architecture. Since the moving speed of the hockey is very fast and the distance within the table for the experimental environment is not large, we consider it in real-time, including the recognition time, the prediction time, and the defender’s movement time. The total of these three times is less than the movement time of hockey (hockey moving distance divided by the fastest puck speed).

In terms of target recognition, we use deep learning models as a recognition method to reduce the impact of environmental brightness on the characteristics of the hockey. We choose YOLO as its algorithm to meet the requirement of real-time.

We also added a neural network-like prediction method, and compared their prediction accuracy and prediction time because hockey may be affected by the friction and levelness of the table to change its linear motion trajectory, in addition to the linear formula combined with the law of reflection for prediction.

In terms of cost and control difficulty, we use stepping motors with linear slides as the move control of the defender, and the stepping motor may lose step during the control process (the motor torque is not enough to drive the defender move), causing the system to make an error in judging the position of the defender’s movement, so it will move the defender as fast as possible without losing step with different control mechanisms.

**Figure 2:**
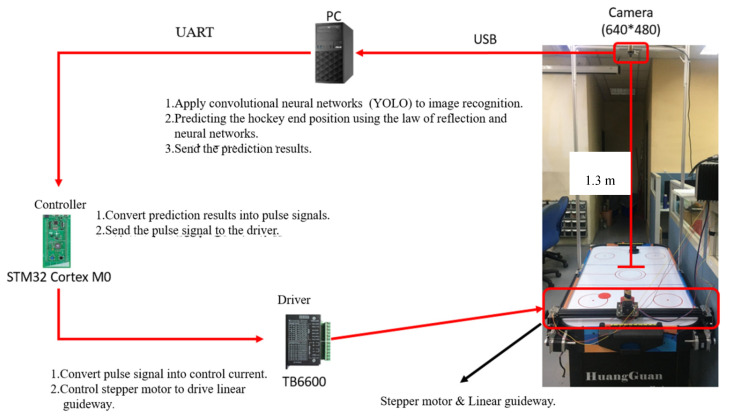
Diagram of system architecture deployment.

The overall system architecture is shown in Figure 2. The camera lens is mounted 1.3 m above the table to get the entire desktop image in real time. It sends real-time images to the PC via USB to do image recognition. The target recognition is performed by the camera capturing the entire air hockey table image and then using a convolutional neural network (YOLO) to detect the position of the hockey puck and the grip of the attacker. Then, the final position of the hockey puck is predicted by the neural network and the reflection law based on the direction of the attacker’s force and the hockey ball’s trajectory. Finally, the prediction result is transmitted to the controller and the stepper motor is controlled to move the defender to block the hockey ball from scoring.

The detail is introduced later for two system implementation methods. It is divided into three main parts, which are the image recognition for identifying the puck, the prediction for computing and predicting the end position of the hockey, and the control for controlling the linear guideway of the stepper motor.

### 3.1. System Implementation—Method 1: Direct Prediction

**Figure 3:**
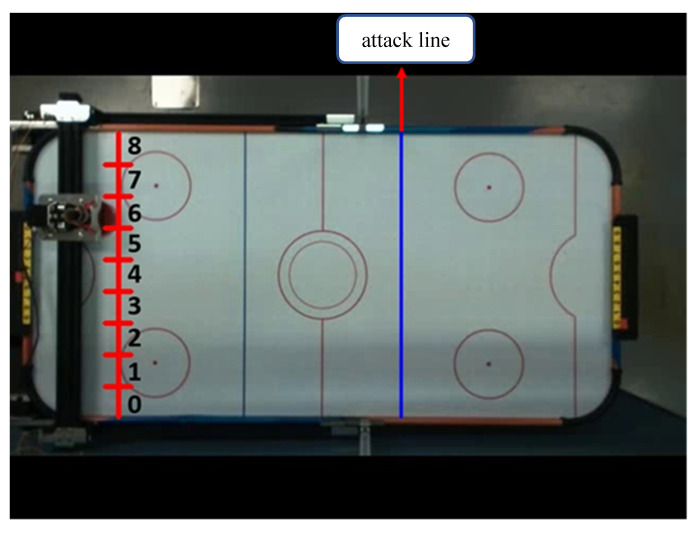
Schematic diagram of defense area of system implementation method 1.

As shown in Figure 3, method 1 is that when the hockey ball passes the attack line, it will predict its end position in the direction of the hockey movement and move the defender to the predicted area. Because the total length of the defensive area is 59 cm and the diameter of the defender is 7.5 cm, the defensive area is divided into nine areas; one area is about 6.6 cm wide.

#### 3.1.1. Image Recognition

**Figure 4:**
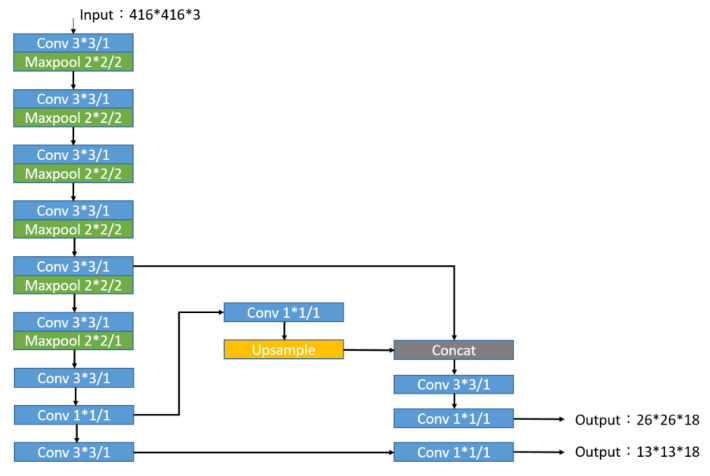
Tiny-YOLOv3 network architecture in method 1.

By testing with the same hardware device (GeForce GTX 1050 Ti, 4GB, NVIDIA Corporation in California, Santa Clara, CA, USA), the recognition speed of YOLOv3 [27] is 15 fps on average, while tiny-YOLOv3 can reach an average of 30 fps. Therefore, tiny-YOLOv3 is used as the target recognition method. The network architecture is shown in Figure 4. The original YOLOv3′s 53-layer convolutional layer is reduced to 13-layer convolutional layers. Except for the last convolutional layer, all convolutional layers are followed by batch normalization to normalize the output data and uses the *LeakyRelu* function that performs a nonlinear transformation on the normalized data. The *LeakyRelu* function is defined as

(1) $$LeakyRelu(x)=\{\begin{array}{cc} \alpha x, & \mathrm{if}\;x<0\\ x, & \mathrm{if}\;x\geq 0 \end{array}$$

where α=0.1.

Since the target category to be identified is only the hockey, the output dimensions of the network architecture can be expressed as 26 × 26 × 3 × (5 + 1) and 13 × 13 × 3 × (5 + 1), where 26 × 26 and 13 × 13 represent the size of the segmented grid. The value of 3 gives the bounding boxes predicted by each grid; 5 includes the size, bounding box position, and credibility score; and 1 is the probability of the hockey.

**Figure 5:**
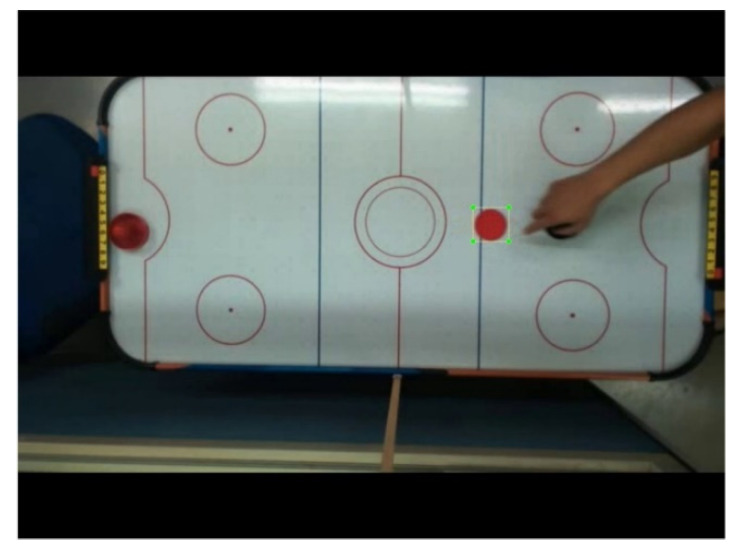
Image of hockey moving slowly.

**Figure 6:**
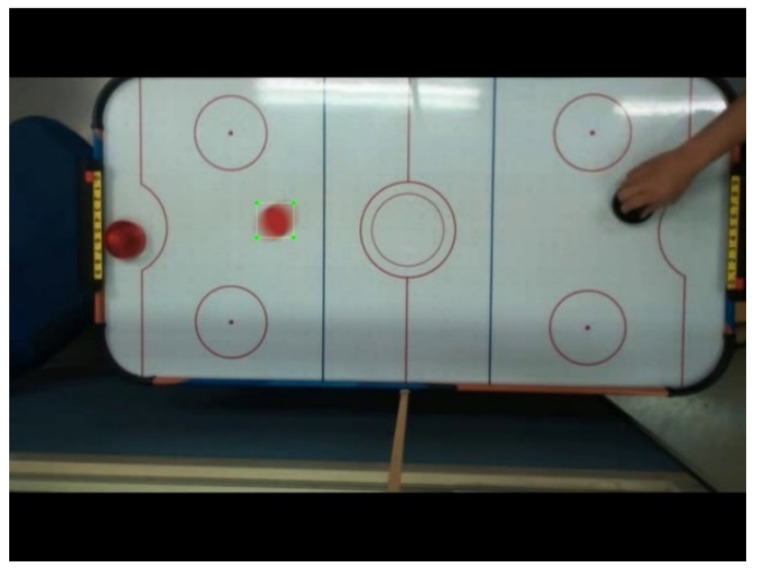
Image of hockey moving fast.

The training data are divided into two different training data groups based on the video footage of the actual game process. One is 250 clear images of hockey moving slowly, as shown in Figure 5 and the other is 250 blurred images of hockey moving fast, as shown in Figure 6—a total of 500 hockey images. The size of the hockey mark of the blurred image is based on the size of the clear hockey mark.

The loss function used in training is the loss function of YOLOv3, and binary classification prediction is made according to whether there is a target in the bounding box (i.e., if there is this category). Target detection and type prediction are calculated by binary cross-entropy.

#### 3.1.2. Prediction Computing

In an ideal situation, the hockey ball moves in a linear motion. However, environmental factors such as table friction and levelness will affect the movement of the hockey. Therefore, we use linear formulas combined with the law of reflection to compare the accuracy of prediction and execution time with the proposed method that uses neural networks.

**Figure 7:**
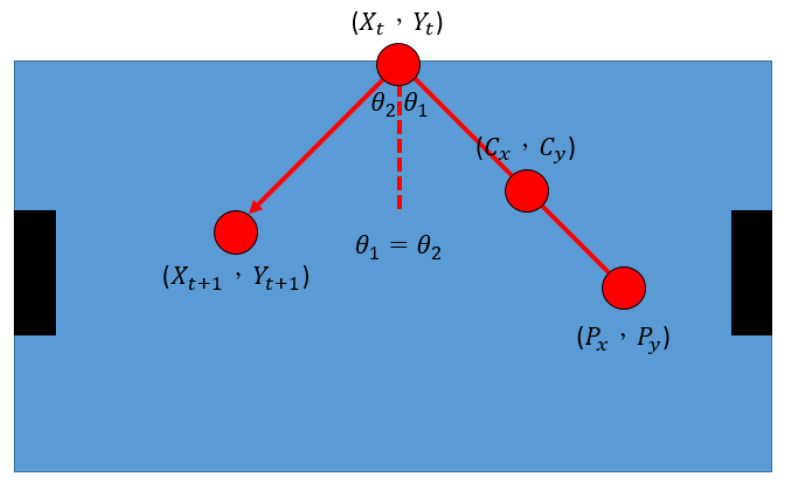
Schematic diagram of linear formula combined with the law of reflection.

**Figure 8:**
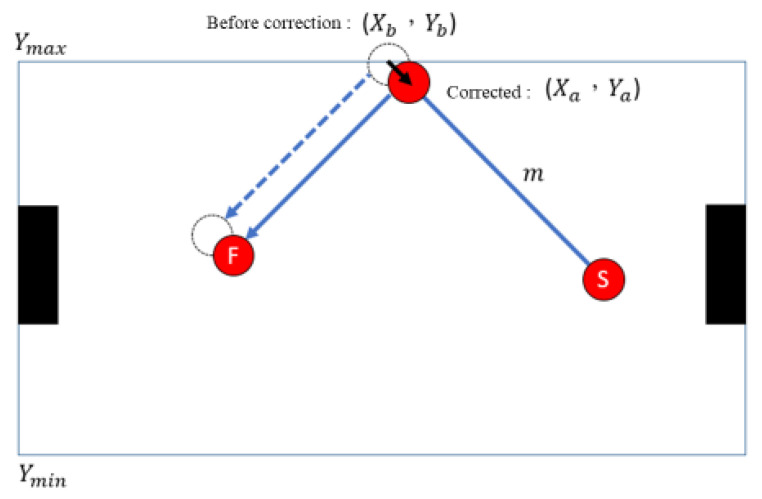
Schematic diagram of correcting impact point.

Using a linear formula combined with the law of reflection to predict the end position is based on the linear formula (before hitting the edge of the table) and the law of reflection (after hitting the edge of the table) to calculate its trajectory, as shown in Figure 7. First, we calculate the trajectory before the collision:

(2) Xt=Cx+(Yt−Cy)(Cy−PyCx−Px)

where $X_{t}$ and $Y_{t}$ represent the final position before hitting the edge of the table; $C_{x}$ and $C_{y}$ represent the hockey center point at the current point in time; and $P_{x}$ and $P_{y}$ represent the hockey center point at the previous point in time. If a collision occurs during the prediction process, then we use the reflection law that the incident angle is equal to the reflection angle to calculate the final coordinate position, as shown in the following formula:

(3) $$Y_{t+1}=-\left(\frac{C_{y}-P_{y}}{C_{x}-P_{x}}\right)(X_{t+1}-X_{t})+Y_{t}$$

where $X_{t+1}$ and $Y_{t+1}$ represent the final position of the set defensive area after hitting the edge of the table. As shown in Figure 8, when calculating the collision point, it is necessary to consider that the hockey puck is a circle instead of a point, so we reverse the actual collision point to correct for the hockey radius by the following two formulas:

(4) $$X_{a}=Y_{b}-\sin(\tan^{-1}(m))$$

(5) $$Y_{a}=\{\begin{array}{cc} Y_{\min}+\cos(\tan^{-1}(m)), & if\;Y_{b}<Y_{\min}\\ Y_{\max}-\cos(\tan^{-1}(m)), & if\;Y_{b}>Y_{\max} \end{array}$$

where $X_{a}$ and $Y_{a}$ are the impact points after correction; $X_{b}$ and $Y_{b}$ are the impact points before correction; $Y_{\min}$ and $Y_{\max}$ represent the upper and lower boundaries, respectively; and *m* is the slope of the current direction of travel.

The neural-network prediction method regards the prediction problem as a classification problem, and divides the final prediction coordinates into nine regions (the width of the game environment is 59 cm and the grip diameter is 7.5 cm). The input data are the coordinates of the three time points and the collision speed between the two points. There are a total of eight input data. Because of the different input values, the input data are normalized first. Here, the minimum maximum value is used. Min–max normalization scales the data to the interval (0, 1), and the output is the area from 0 to 8. Referring to the network architecture mentioned in the neural network-based air hockey robot strategy forecast published by Jung Il Park, Chad B. Partridge, and Mark W. Spong in 2001, their experiment included three different architectures:

Architecture I. input layer → hidden layer (128) → output layer;
Architecture II. input layer → hidden layer (2048) → hidden layer (128) → output layer;
Architecture III. input layer → hidden layer (2048) → hidden layer (1024) → hidden layer (128) → output layer

We also compare the prediction accuracy and execution time. The excitation functions are all sigmoid, and the final output layer uses the softmax function to normalize the output value to the (0, 1) interval, which represents the probability of outputting each area from 0 to 8, and the sum is 1. The maximum value is the prediction area, the loss function for cross entropy, and the learning optimization algorithm used is adam.

#### 3.1.3. Control

A stepper motor, also known as a step motor or stepping motor, is a brushless DC electric motor that divides a full rotation into a number of equal steps. The motor’s position can then be commanded to move and hold at one of these steps without any position sensor for feedback (an open-loop controller), as long as the motor is carefully sized to the application with respect to torque and speed. Motor resonance frequency can be calculated from the formula:

(6) $$f=\frac{100}{2\pi }\sqrt{\frac{2pM_{h}}{J_{r}}}$$

where *M_h_* is holding torque (N·m), *p* is number of pole pairs, and *J_r_* is rotor inertia (kg·m^2^).

**Figure 9:**
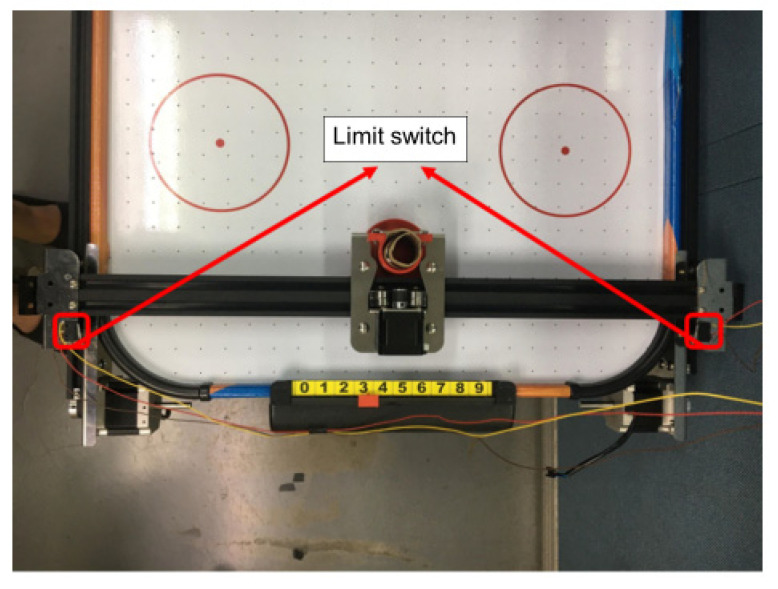
Limit switch installation diagram.

Since the stepping motor is controlled by a pulse signal, it needs to be initialized to obtain the number of pulses corresponding to the moving distance. The initialization process is to set the limit distance of the defender’s movement through the mechanical limit switch. As shown in Figure 9, the limit switch is installed to the left and right sides of the slide rail. First, the stepping motor controls movement of the defender to the left. When the limit switch is reached, then it starts to move in the opposite direction and records the number of pulses until it hits the right limit switch, thereby calculating the number of pulses required to move a defensive area, and moves the defender to the center of the track after the initialization is completed, that is, defensive area four.

**Figure 10:**
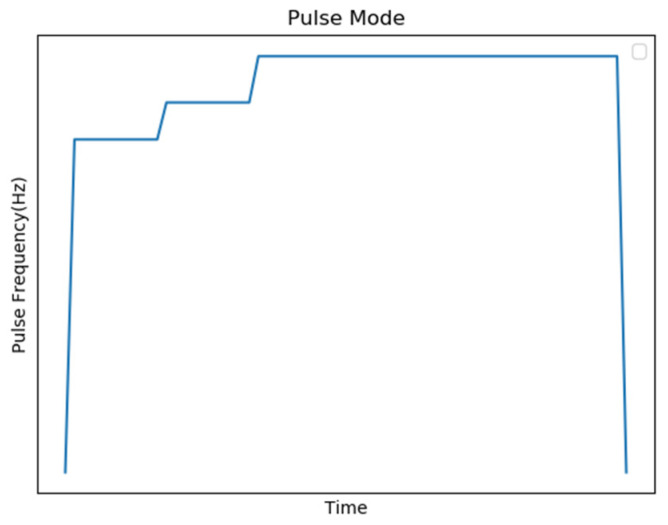
Schematic diagram of stepping motor control mechanism.

When the speed of the stepping motor is slow, the coil current has enough time to reach the maximum value and the output torque is larger. On the contrary, when the speed increases, the pulse signal changes rapidly, so that the coil current weakens and the output torque decreases. The movement of the defender must meet the conditions of the high-speed hockey movement, so the original pulse selection part is initialized to control the motor with the highest pulse frequency (2500 Hz), but through experiments, it was found that controlling the motor at this frequency is prone to insufficient torque. In the situation when the phenomenon of synchronization loss occurs, although the controller sent a control signal, the defender did not move, causing the system to make an error in the position judgment, which makes it impossible to block the hockey in real time and needs to be reinitialized. In order to solve this problem, the frequency of the start pulse signal is changed to a gradual increase instead of directly using the highest speed frequency pulse to control the motor, as shown in Figure 10.

### 3.2. System Implementation—Method 2: Two-Stage Prediction

This method divides the prediction of the hockey end position into a preliminary and final prediction. The preliminary prediction is to use the neural network to roughly estimate the end position of the hockey through the direction of the attacker’s force before the hockey is hit by the attacker’s grip; the final prediction is to predict the end position of the hockey by the linear formula combined with the law of reflection.

#### 3.2.1. Image Recognition

**Figure 11:**
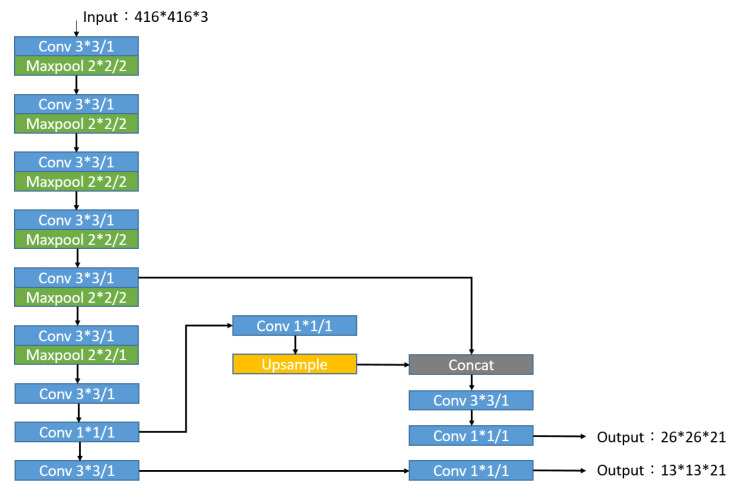
Tiny-YOLOv3 network architecture.

Due to the addition of preliminary predictions, there are two types of targets that need to be identified, hockey and the attacker’s grip, and the output dimension becomes 26 × 26 × 3 × (5 + 2) and 13 × 13 × 3 × (5 + 2), where 26 × 26 and 13 × 13 represent the size of the segmented grid. The value of 3 gives the bounding boxes predicted by each grid; 5 includes the size, bounding box position, and credibility score; and 2 is the probability of hockey and attacker’s grip. The network architecture is shown in Figure 11.

Since both hockey and the attacker have only one, the bounding box with the highest credibility score is selected as the prediction result. The loss function is the same as the loss function of tiny-YOLOv3 mentioned in method 1.

#### 3.2.2. Prediction Computing

**Figure 12:**
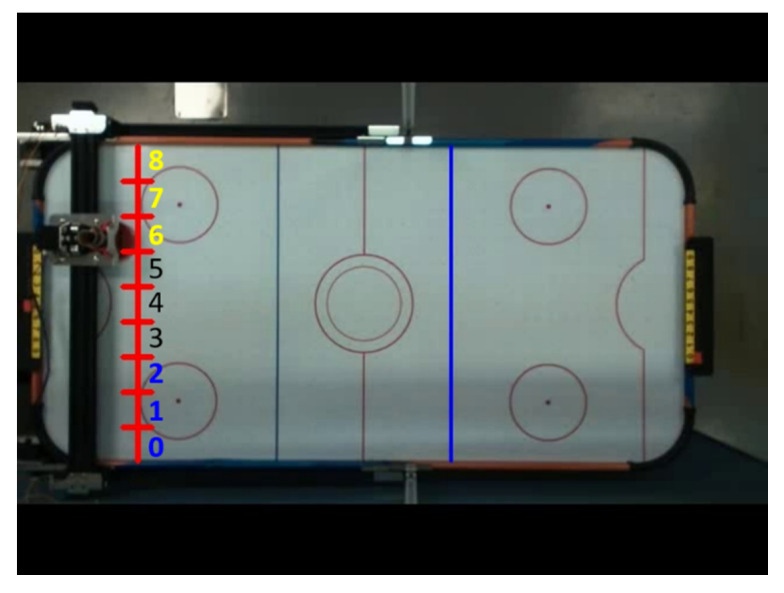
Schematic diagram of preliminary predicted moving area.

The prediction of the hockey end position is divided into preliminary prediction and final prediction. The preliminary prediction uses the direction of force applied by the attacker before hitting the ball as the basis for rough estimation of the hockey end position. It is carried out when the distance between the attacker’s grip and the stick is less than 10 cm. The results of the prediction are divided into three areas, as shown in Figure 12, which are the upper half of the defense area (area 6, 7, 8), the lower half (area 0, 1, 2), and the center area (area 3, 4, 5). If it is the upper half, the defender moves to area 6; if it is the lower half, it moves to area 2; if the prediction result is in the center area, the defender stays in area 4.

Preliminary predictions will be conducted with a neural network-like method to compare the accuracy of predictions of different network architectures, and the architecture with the highest accuracy selected as a neural network architecture such as this prediction method. This system regards predicting the final area of hockey as a classification problem, and uses an open source tool, Auto-Keras [28,29,30], to search the network architecture.

**Figure 13:**
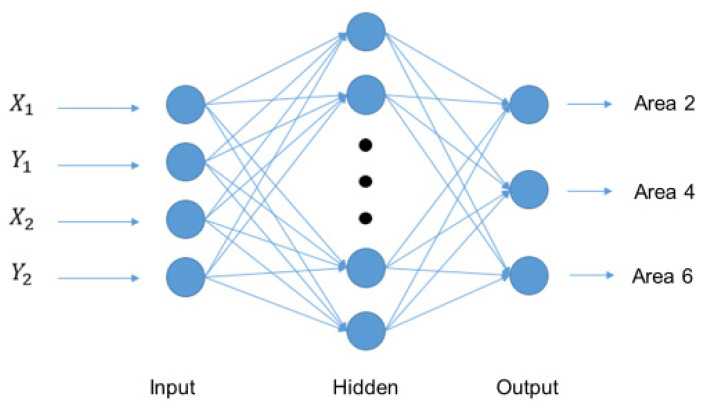
Network architecture of data set 1.

**Figure 14:**
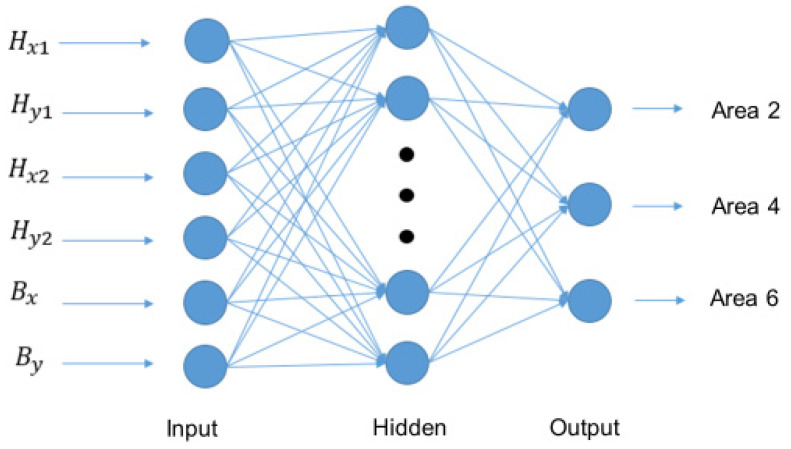
Network architecture of data set 2.

We use the two different input data sets to search for suitable network architecture and compare its prediction accuracy. Data set 1 is 1000 pieces of data of the two grip positions before hitting the hockey. As shown in Figure 13, $X_{1}$, $Y_{1}$, $X_{2}$, $Y_{2}$ represent the position of the two-point grip. Data set 2 is the two-point grip position before hitting the hockey ball, plus the 1000 data of the current position of the hockey ball. As shown in Figure 14, $H_{x1}$, $H_{y1}$, $H_{x2}$, $H_{y2}$ represent the two-point grip position and $B_{x}$, $B_{y}$ represent the hockey position. The operation of neural network is as follows. A neuron with label $y_{j}$ receives an input $x_{i}$ such as $X_{1}$, $Y_{1}$, $X_{2}$, $Y_{2}$, $H_{x1}$, $H_{y1}$, $H_{x2}$, $H_{y2}$ from predecessor neurons by a connection assigned a weight $w_{ji}$.

(7) $$y_{j}=\sum_{i}w_{ji}x_{i}=W_{j}^{T}X$$

where $W_{j}={\left[\begin{array}{ccc} \cdot \cdot \cdot & w_{ji} & \cdot \cdot \cdot \end{array}\right]}^{T}$ and $X={\left[\begin{array}{ccc} \cdot \cdot \cdot & x_{i} & \cdot \cdot \cdot \end{array}\right]}^{T}$. The error $e_{j}$ between real output $d_{j}$ and $y_{j}$ is then rewritten as

(8) $$e_{j}=d_{j}-y_{j}=d_{j}-W_{j}^{T}X$$

or equivalently

(9) $$\frac{1}{2}e_{j}^{2}=\frac{1}{2}{\left(d_{j}-W_{j}^{T}X\right)}^{2}$$

The total error is

(10) $$\frac{1}{2}e^{2}=\frac{1}{2}\sum_{j}e_{j}^{2}=\frac{1}{2}\sum_{j}{\left(d_{j}-W_{j}^{T}X\right)}^{2}$$

that implies

(11) $$\frac{1}{2}\frac{\partial e^{2}}{\partial W_{j}}=-\sum_{j}(d_{j}-W_{j}^{T}X)X=-eX$$

By introducing a parameter η called the learning rate, the new weighting $W_{j}^{new}$ is obtained by

(12) $$W_{j}^{new}=W_{j}-(-\eta eX)=W_{j}+\eta eX$$

This expression means that each input will correspond to a different weight, and the output is multiplied by the weight to calculate the input of the next layer, and the weight is continuously updated until convergence, which finally gives the continuous output.

The output of the two data sets are the respective probabilities of regions 2, 4, and 6, and because of the different input values, the input data will be normalized first. Here, the minimum and maximum normalization (min–max normalization) scale the data to the interval (0,1), and different network architectures and different excitation functions are experimented with. The final output layer uses the softmax function to normalize the output value to the (0, 1) interval, which provides a representation of the probability of the three regions. The sum is one, and the maximum value is taken as the prediction region, the loss function is cross entropy, and the learning optimization algorithm used is adam.

The final prediction uses the linear formula mentioned in method 1 combined with the law of reflection to predict the final zone position of the hockey when the hockey passes the center line.

#### 3.2.3. Control

The control mechanism of initializing a stepper motor is the same used in method one. The movement after initialization will be moved according to the two prediction results. The movement based on the preliminary prediction result is called the first stage movement, and the movement based on the final prediction result is called the second stage movement.

Since the movement is divided into two stages, there will be two different movement conditions. The first situation is that when the movement direction of the second stage control is the same as that of the first stage, the starting torque of the second stage control only needs to be the same as the torque of the first stage control to drive the movement of the defender; therefore, it is at the pulse frequency. The selection of this is the same as the pulse frequency of the first stage.

The second situation is that the movement direction of the second stage control is different from that of the first stage. Since the interval between the two stages of control is quite short, the movement of the second stage immediately after the end of the first stage will cause the motor to move in the first stage. After the stage control operation is completed, the discharge is not complete. If the motor is controlled with the same pulse frequency as the first stage control, the motor will not be able to drive the second stage operation due to insufficient torque, and the motor will lose step. Therefore, two methods are proposed. Different stepping motor control mechanisms are used, and their out-of-step conditions and the time spent moving are compared. Therefore, two different stepping motor control mechanisms are proposed and compared for the out-of-synchronization situation and the time taken to move.

**Figure 15:**
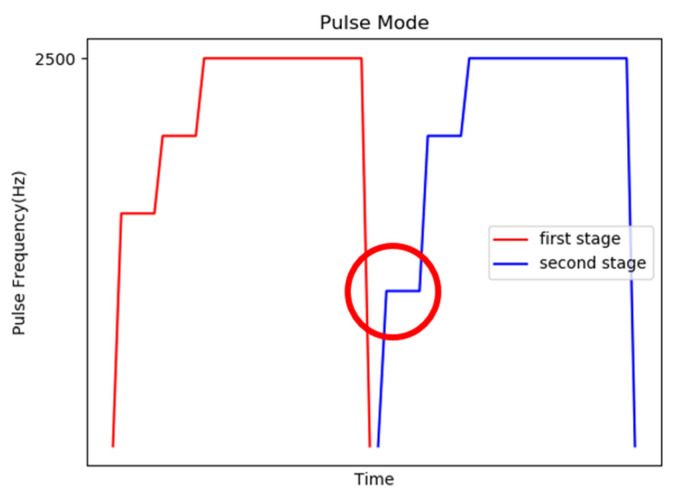
Schematic diagram of the first control mechanism.

The first control mechanism is to start slowly and then run at a constant speed and then stop, and when the moving directions of the two stages are different, the start pulse frequency of the second stage is reduced to increase the torque, as shown in Figure 15, to smoothly control the motor.

**Figure 16:**
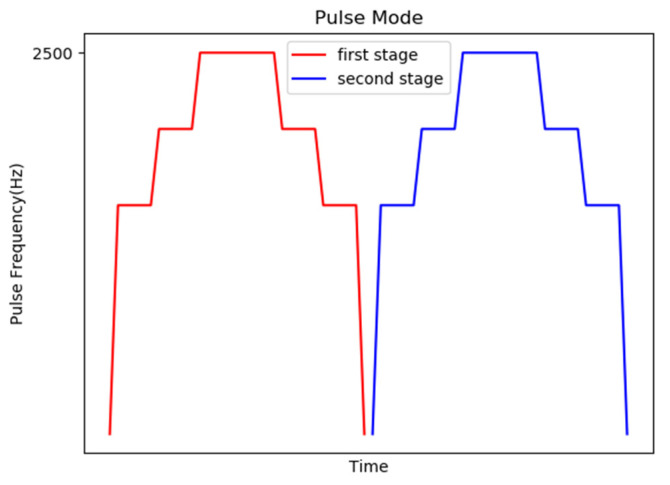
Schematic diagram of the second control mechanism.

The second control mechanism is to change the two-stage motor control to a slow start and a constant speed operation and then a slow stop for control, as shown in Figure 16.

## 4. Experimental Environment and Results

### 4.1. Experimental Environment

**Figure 17:**
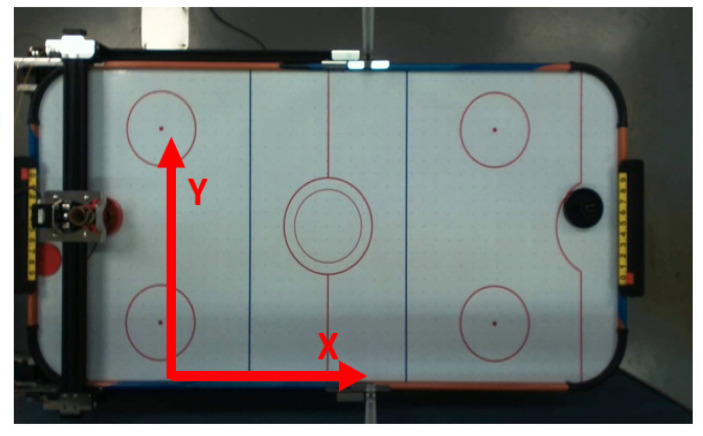
Air hockey table.

The air hockey table and related parameters are shown in Figure 17 and Table 1.

**Figure 18:**
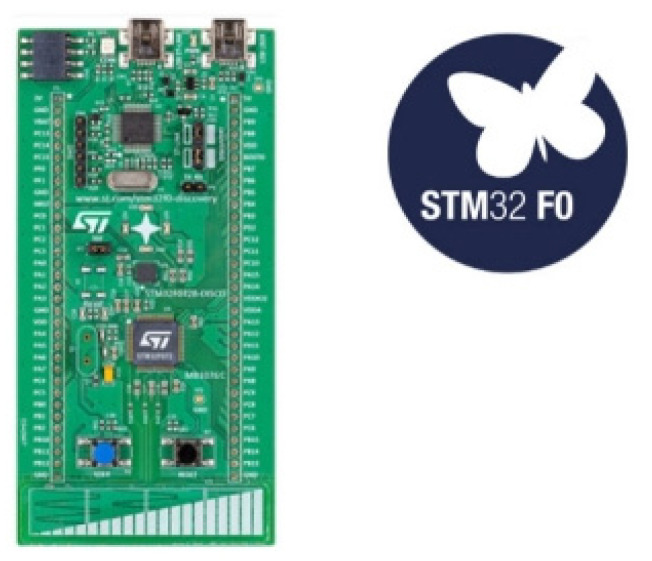
32F072BDISCOVERY based on Cortex-M0 as the core.

**Figure 19:**
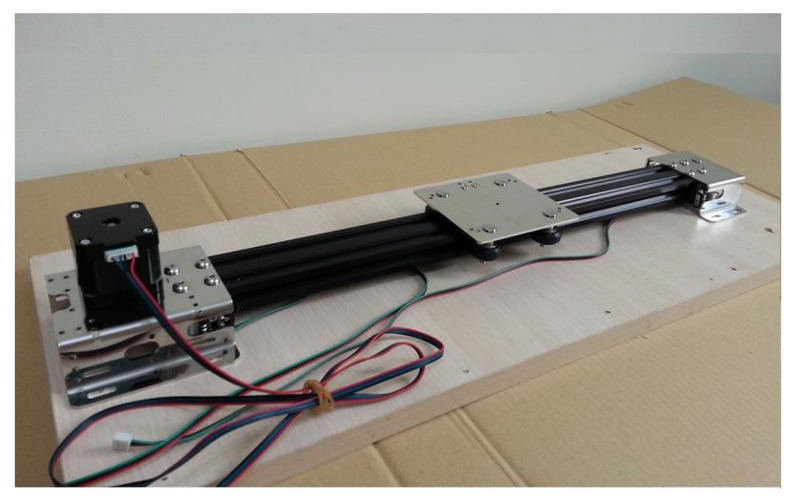
Ball-type linear slide.

**Figure 20:**
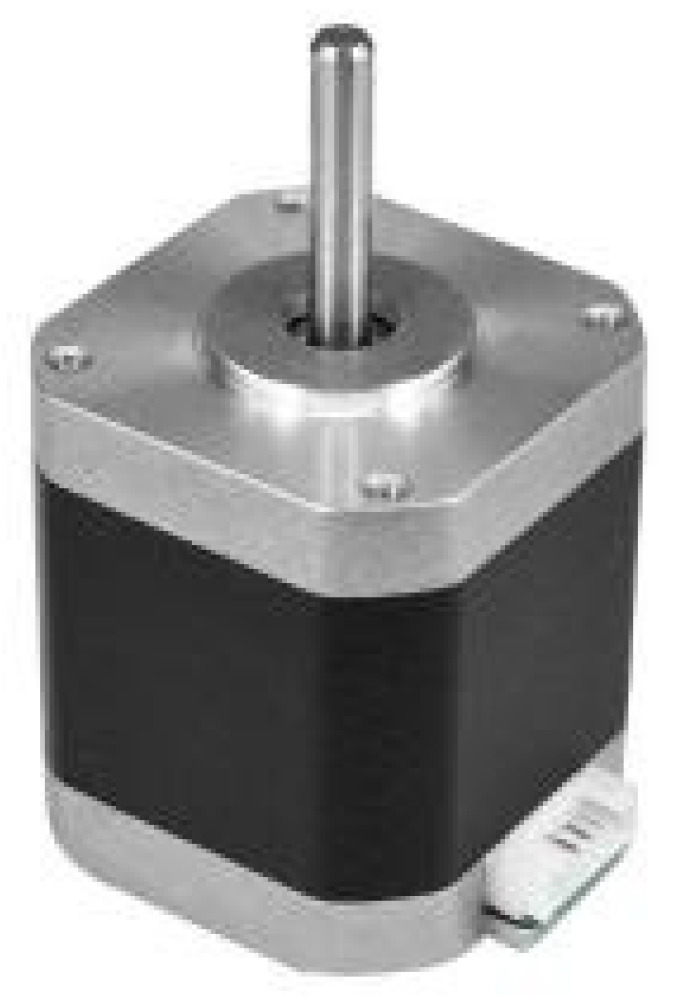
Stepping motor 42BYGH4417 NEMA 17.

STM32 is a 32-bit microcontroller launched by STMicroelectronics and is equipped with a variety of communication interfaces: I2C, SPI, UART, etc., which can be connected to sensors or used as independent controllers according to needs. The development environment of STM32 uses the C language, and there are many different series of choices according to the needs. In this work, we chose 32F072BDISCOVERY based on Cortex-M0 as the core, as shown in Figure 18, with four sets of UART, two sets of SPI, two sets of I2C, and other interfaces. The linear slide used in this work is a ball-type linear slide, as shown in Figure 19. The stepping motor in this work adopts the model 42BYGH4417 NEMA 17, as shown in Figure 20, which is a two-phase, four-wire motor with an 8 mm screw rod diameter and a minimum movement about 0.001 m.

In addition, the pixel of the camera is set to 640 × 480 and the operating system of the personal computer (PC) is Windows 10. The PC hardware equipment is listed in Table 2.

### 4.2. Experimental Results

The following experimental results are presented: identification effect of the self-trained tiny-YOLOv3, the accuracy and execution time of the hockey end position prediction of the different methods, the frequency pulse combination of different modes of stepping motors, the defender’s movement control in method 2, the fastest ball speed that the system can block after the actual test, and 100 actual game results.

#### 4.2.1. Identification Effect of the Self-Trained Tiny-YOLOv3

Because the background of the training data is similar, the number of training times is set to 1000, and the IoU and credibility score thresholds are set to 0.5 after the model converges, and the identification of 100 actual game images is tested. The pixel setting is 640 × 480. The average position and recognition speed is 30 frames per second. All the targets in the test data include hockey and the attacker’s grip. The classification results and positioning errors are shown in Table 3 and Table 4.

#### 4.2.2. Accuracy and Execution Time of the Hockey and Position Prediction for Different Methods

In preliminary prediction of method 2, as shown in Table 5, we set up eight models with different parameters to train neural networks, including 1000 training data, 500 test data, and 10,000 training times. The input layer and hidden layer in the model all use the same excitation function for nonlinear conversion. In the final prediction of method 2, the final position of the hockey ball after passing through different designated areas was predicted by a linear formula combined with the reflection law. A total of 500 data were tested. As shown in Table 6, the experimental results show that the hockey has a higher accuracy rate after passing the center line. Finally, accuracy and execution time of the hockey and position prediction for different methods are listed in Table 7. Since system implementation method 2 has best accuracy and execution time of the hockey and position prediction, we adopt system implementation method 2 to be the main method for later experiments.

#### 4.2.3. Defender’s Movement Control in Method 2

The stepping motor control mechanism of method 2 is divided into two stages with the same moving direction and two stages with different moving directions to compare the moving time under different control mechanisms. The instructions follow.

**Figure 21:**
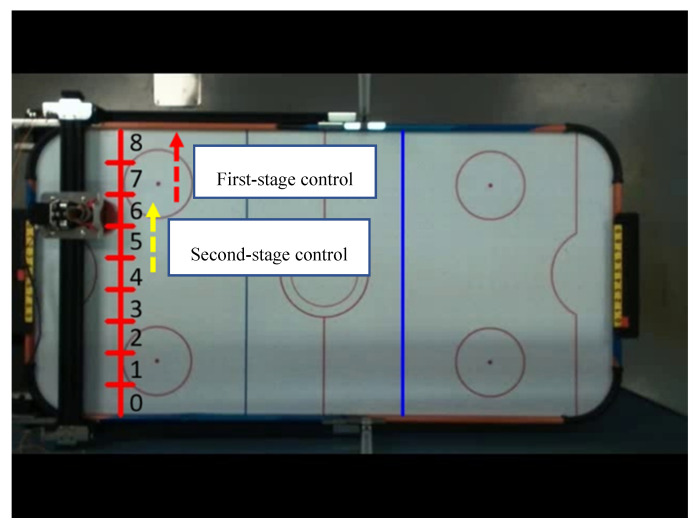
Diagram of the same moving direction.

**Figure 22:**
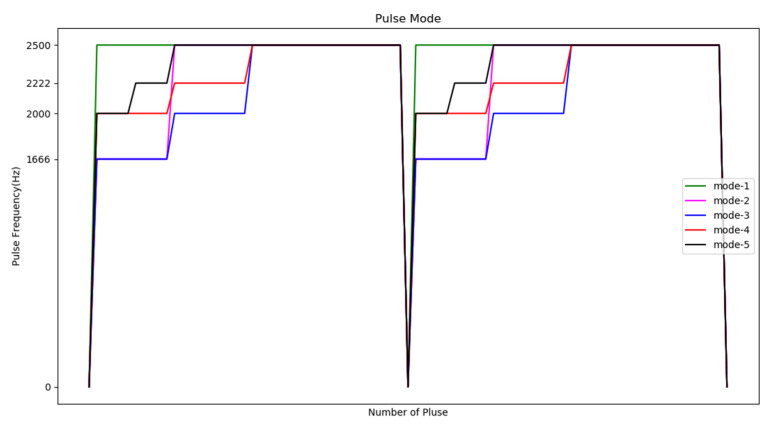
Time distribution diagram of pulse frequency.

As shown in Figure 21, the control mechanism of two stages with the same moving direction (for example, 4 → 6 → 8) is Start slowly → Constant speed operation → Stop. We experimented with five different progressive pulse frequency combinations, as shown in Figure 22 and Table 8. The movement time in Table 8 is the total time (unit: second) to move the four defensive areas, and T represents the number of pulses required to move one area. We used the fastest mode five that does not cause the motor to lose step and the fastest moving speed as the pulse frequency to control the motor when the direction is the same.

**Figure 23:**
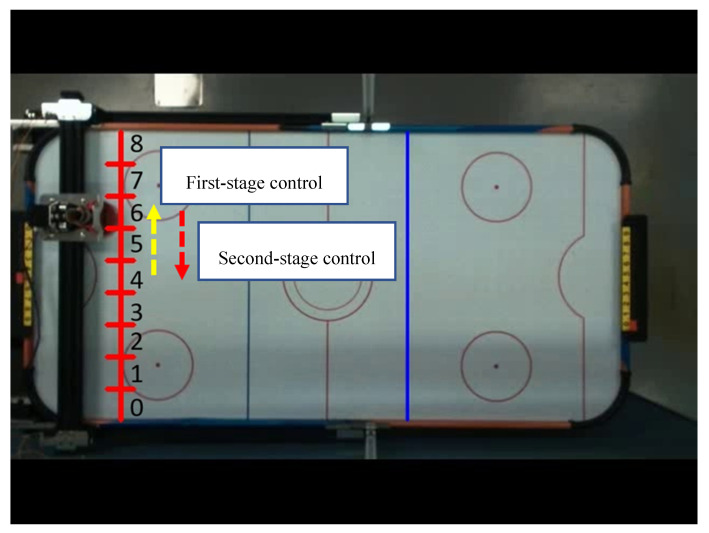
Diagram of different moving directions.

**Figure 24:**
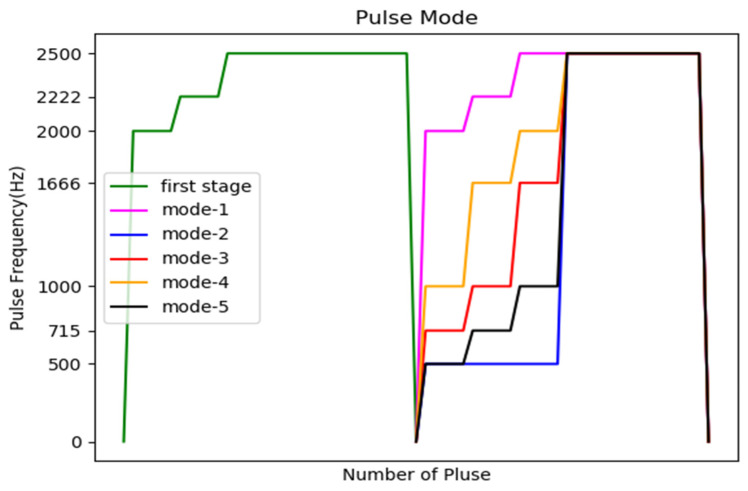
Time distribution diagram of pulse frequency in the first control mechanism.

**Figure 25:**
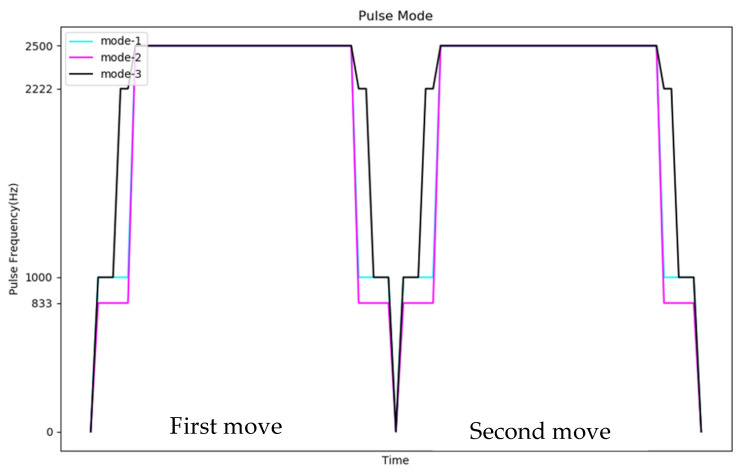
Time distribution diagram 1 of pulse frequency in the second control mechanism.

**Figure 26:**
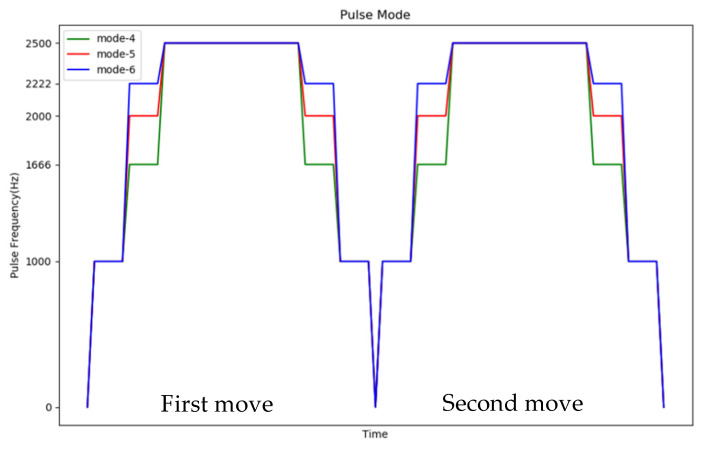
Time distribution diagram 2 of pulse frequency in the second control mechanism.

As shown in Figure 23, there are two control mechanisms for two stages with different moving directions (for example, 4 → 6 → 4). The first control mechanism is Start slowly → Constant speed operation. In the second stage, it started with a lower frequency pulse, and experimented with five different progressive pulse frequency combinations, as shown in Figure 24. The second control mechanism is Start slowly → Constant speed operation → Stop slowly. We also experimented with six different progressive pulse frequency combinations, as shown in Figure 25 and Figure 26. The experimental results are shown in Table 9 and Table 10. The movement time in Table 9 and Table 10 is the total time (unit: second) to move the four defense areas, and T represents the number of pulses required to move one area. After comparison, it is found that mode 5 of control mechanism 1 will not cause the motor to lose step and move the fastest. Mode 5 of control mechanism 1 is used as the pulse frequency to control the motor when the direction is opposite.

#### 4.2.4. The Fastest Ball Speed That the System Can Block after the Actual Test, and 100 Actual Game Results

In order to successfully block the hockey, the sum of the recognition time, prediction time, and defender movement time must be less than the hockey movement time (hockey movement distance/fastest ball speed). The hockey movement distance is from the midpoint of the attack line to the farthest defense; i.e., the distance of the area (area 8/area 0). The experimental results are shown in Table 11.

The actual game prediction method is to use method two and model 5 as the neural network architecture for preliminary prediction. After the hockey passes the center line, the final prediction is made by using linear formulas and the law of reflection. The number of games is 100. The fastest ball speed does not exceed 1.15 m/s. The statistical results are shown in Table 12. The actual blocking probability in most areas is much lower than the final prediction accuracy. The reason is that the initial prediction error caused the defender to move the farthest area by two. The reason for the increase to six and the actual blocking probability in area 5 being greater than the predicted correct rate is because the hockey is recorded as a blocking during the movement success, although the defender move is based on the result of the wrong prediction.

## 5. Conclusions

From the experimental results, it is found that the method of prediction using the law of reflection can block the fast-moving hockey instantly when the hockey speed does not exceed three meters per second. When predicting with the direction of the force, it is found that when the player uses wrist movement to swing, it will reduce the accuracy of the predictions. In the future, player gestures will be added as neural networklike inputs to improve the accuracy of the predictions, and control of return strategies will be added.

## Figures and Tables

**Table 1:** Experimental environment.

Table length	109 cm
Table width	59 cm
Goal width	19 cm
Air hockey ball diameter	6.3 cm
Plstic handle diameter	7.5 cm

**Table 2:** Hardware equipment.

Memory	24 GB
CPU	Intel i5-6500 cpu @ 3.20 GHz
GPU	Nvidia Geforce GTX 1050Ti 4 GB

**Table 3:** The classification results.

	Positive	Negative
True	94	-
False	0	6
Average center positioning error	2.21 (pixel) → 0.48 (cm)	
Maximum center positioning error	4.03 (pixel) → 0.88 (cm)	

**Table 4:** Attacker’s handle recognition result.

	Positive	Negative
True	100	-
False	0	0
Average center positioning error	2.71 (pixel) → 0.59 (cm)	
Maximum center positioning error	4.71 (pixel) → 1.03 (cm)	

**Table 5:** Eight models with different parameters to train neural networks.

	Model l	Model 2	Model 3	Model 4	Model 5	Model 6	Model 7	Model 8
Input layer	Two-point grip position				Two-point grip position		Hockey position	
Hidden layer 1	512	512	512	512	512	512	512	512
Hidden layer 2	256	256	256	256	256	256	256	256
Hidden layer 3			64	64			64	64
Output layer	Probability of zone 2, 4, 6, respectively							
Excitation function	Sigmoid	ReLu	Sigmoid	ReLu	Sigmoid	ReLu	Sigmoid	ReLu
Loss function	Categorical Cross Entropy							
Prediction accuracy	0.54	0.57	0.44	0.59	0.80	0.75	0.79	0.55

**Table 6:** Prediction accuracy and execution time in method 2.

Final Prediction (Linear Formula Combined with the Reflection Law)	Accuracy	Execution Time (Millisecond)
Hockey prediction after hockey passes the attack line	0.79	0.04
Hockey prediction after passing the center line	0.84	0.04

**Table 7:** Accuracy and execution time of the hockey end position prediction of different methods.

Method	Accuracy	Execution Time
Linear formula combined with the law of reflection	79%	0.04 ms
System implementation method 1	49%	2.5 ms
System implementation method 2	84%	0.04 ms

**Table 8:** Pulse mode.

	First Move	Second Move	Time	Lose Step
Mode 1	2500 Hz × 2T	2500 Hz × 2T	0.664	yes
Mode 2	1666 Hz × 10 → 2500 Hz × 10 → 2500 Hz × (2T − 20)	1666 Hz × 10 → 2500 Hz × 10 → 2500 Hz × (2T − 20)	0.674	no
Mode 3	1666 Hz × 10 → 2000 Hz × 10 → 2500 Hz × (2T − 20)	1666 Hz × 10 → 2000 Hz × 10 → 2500 Hz × (2T − 20)	0.672	no
Mode 4	2000 Hz × 10 → 2222 Hz × 10 → 2500 Hz × (2T − 20)	2000 Hz × 10 → 2222 Hz × 10 → 2500 Hz × (2T − 20)	0.670	no
Mode 5	2000 Hz × 5 → 2222 Hz × 5 → 2500 Hz × (2T − 10)	2000 Hz × 5 → 2222 Hz × 5 → 2500 Hz × (2T − 10)	0.668	no

**Table 9:** Pulse mode in the first control mechanism.

	First Move	Second Move	Time	Lose Step
Mode 1	2000 Hz × 5 → 2222 Hz × 5 → 2500 Hz × (2T − 10)	2000 Hz × 5 → 2222 Hz × 5 → 2500 Hz × (2T − 10)	0.668	yes
Mode 2	2000 Hz × 5 → 2222 Hz×5 → 2500 Hz × (2T − 10)	500 Hz × 15 → 2500 Hz × (2T − 15)	0.696	no
Mode 3	2000 Hz × 5 → 2222 Hz×5 → 2500 Hz × (2T − 10)	715 Hz × 5 → 1000 Hz×5 → 1666 Hz × 5 → 2500 Hz × (2T − 15)	0.683	yes
Mode 4	2000 Hz × 5 → 2222 Hz×5 → 2500 Hz × (2T − 10)	1000 Hz × 5 →1666 Hz×5 → 2000 Hz × 5 → 2500 Hz × (2T − 15)	0.677	yes
Mode 5	2000 Hz × 5 → 2222 Hz×5 → 2500 Hz × (2T − 10)	500 Hz × 5 → 715 Hz × 5 → 1000 Hz × 5 → 2500 Hz × (2T − 15)	0.690	no

**Table 10:** Pulse mode in the second control mechanism.

	Pulse of Start	Constant-Speed Pulse	Deceleration Pulse	Time	Lose Step
Mode 1	1000 Hz × 5	2500 Hz × (2T − 10)	1000 Hz × 5	0.690 s	yes
Mode 2	833 Hz × 5	2500 Hz × (2T − 10)	833 Hz × 5	0.698 s	no
Mode 3	1000 Hz × 3 → 2222 Hz × 2	2500 Hz × (2T − 10)	2222 Hz × 2 → 1000 Hz × 3	0.691 s	yes
Mode 4	1000 Hz × 5 → 1666 Hz × 5	2500 Hz × (2T − 20)	1666 Hz × 5 → 1000 Hz × 5	0.695 s	no
Mode 5	1000 Hz × 5 → 2000 Hz × 5	2500 Hz × (2T − 20)	2000 Hz × 5 → 1000 Hz × 5	0.693 s	no
Mode 6	1000 Hz × 5 → 2222 Hz × 5	2500 Hz × (2T − 20)	2222 Hz × 5 → 1000 Hz × 5	0.692 s	no

**Table 11:** The fastest ball speed that the system can block after the actual test.

Implementation Method	Ideal Speed	Speed in Experiment
System implementation method 1	0.96 m/s	0.88 m/s
System implementation method 2	1.11 m/s	1.15 m/s

**Table 12:** One hundred actual game results.

Item Area	0	1	2	3	4	5	6	7	8
Distribution probability by area	0.2	0.18	0.14	0.08	0.05	0.05	0.22	0.18	0.08
Accuracy of preliminary prediction	0.79			0.77			0.79		
Accuracy of final prediction	1	0.83	0.81	0.87	0.8	0.8	0.81	0.77	0.75
Actual blocking probability	0.5	0.55	0.71	0.75	0.8	1	0.72	0.61	0.37
Average blocking rate	0.67								
